# Abnormal left ventricular subendocardial perfusion and diastolic function in women with obesity and heart failure and preserved ejection fraction

**DOI:** 10.1007/s10554-022-02782-x

**Published:** 2023-01-06

**Authors:** Roshanak Markley, Marco Giuseppe Del Buono, Virginia Mihalick, Alexander Pandelidis, Cory Trankle, Jennifer H. Jordan, Kevin Decamp, Chris Winston, Salvatore Carbone, Hayley Billingsley, Andrew Barron, Georgia Thomas, Benjamin Van Tassell, W. Gregory Hundley, Peter Kellman, Antonio Abbate

**Affiliations:** 1 VCU Pauley Heart Center, Division of Cardiology, Department of Internal Medicine, Virginia Commonwealth University, PO Box 980036, 23219 Richmond, VA USA; 2 Department of Cardiovascular and Thoracic Sciences, Fondazione Policlinico Universitario A. Gemelli IRCCS, Catholic University of the Sacred Heart, Rome, Italy; 3 Department of Biomedical Engineering, Virginia Commonwealth University, Richmond, VA USA; 4 Department of Radiology, Virginia Commonwealth University, Richmond, VA USA; 5 Department of Kinesiology & Health Sciences, College of Humanities & Sciences, Virginia Commonwealth University, Richmond, VA USA; 6 C. Kenneth and Diane Wright Center for Clinical and Translational Research, Virginia Commonwealth University, Richmond, VA USA; 7 Department of Pharmacotherapy and Outcome Sciences, Virginia Commonwealth University, Richmond, VA USA; 8 National Heart, Lung, and Blood Institute, National Institutes of Health, Bethesda, MD USA

**Keywords:** Microvascular dysfunction, Heart failure with preserved ejection fraction, Quantitative myocardial perfusion mapping

## Abstract

**Purpose:**

– Coronary microvascular dysfunction (CMD) is common in patients with heart failure with preserved ejection fraction (HFpEF) and obesity. Stress cardiovascular magnetic resonance (CMR) has been proposed as a non-invasive tool for detection of CMD. The aim of this study was to determine relationship between CMD and diastolic function in patients with HFpEF using a novel CMR technique.

**Methods:**

– Patients with obesity and HFpEF without epicardial coronary artery disease (CAD) underwent Doppler echocardiography to measure diastolic function, followed by vasodilator stress CMR, using a single bolus, dual sequence, quantitative myocardial perfusion mapping to measure myocardial blood flow (MBF) at rest and at peak hyperemia. With this, myocardial perfusion reserve (MPR), global stress endocardial-to-epicardial (endo:epi) perfusion ratio, and total ischemic burden (IB, defined as myocardial segments with MBF < 1.94 mL/min/g) were calculated. Results are reported as median and interquartile range.

**Results:**

– Nineteen subjects were enrolled (100% female, 42% Black). Median age was 64 [56–72] years. Global stress MBF was 2.43 ml/min/g [2.16–2.78] and global myocardial perfusion reserve (MPR) was 2.34 [2.07–2.88]. All had an abnormal subendocardial perfusion with an endo:epi of less than 1 (0.87 [0.81–0.90]). Regional myocardial hypoperfusion was detected in 14 (74%) patients with an IB of 6% [0-34.4]. Endo:epi ratio significantly correlated with IB (R=-0.510, p = 0.026) and measures of diastolic function (R = 0.531, p = 0.019 and R=-0.544, p = 0.014 for e’ and E/e’ respectively).

**Conclusion:**

– Using a novel quantitative stress CMR myocardial perfusion mapping technique, women with obesity and HFpEF were found to have patterns of abnormal subendocardial perfusion which significantly correlated with measures of diastolic dysfunction.

## Introduction

Heart failure with preserved ejection fraction (HFpEF) is a heterogenous clinical syndrome accounting for approximately one-half of the total HF patients [1, 2]. Obesity is a common comorbidity in patients with HFpEF [3]. There is an emerging concept of coronary microvascular dysfunction (CMD) driven by obesity and the associated metabolic risk that plays a central role in myocardial fibrosis and cardiomyocyte stiffening and clinical HFpEF [4, 5]. Women are more likely to have CMD than men, and studies have demonstrated that women with ischemic symptoms without obstructive coronary disease and preserved EF have higher mortality and HF hospitalization [6, 7]. Observational studies using invasive or non-invasive functional testing support the premise that CMD is common in patients with HFpEF and that the presence of both CMD and diastolic dysfunction is associated with a markedly increased risk of future HFpEF hospitalization [8, 9]. Current diagnostic pathways to diagnose CMD require the assessment of epicardial coronary anatomy and invasive physiological assessment of the microvasculature’s response to both vasodilator (e.g. adenosine) and provocative challenges (e.g. acetylcholine) [10, 11]. Stress perfusion cardiac magnetic resonance (CMR) has become a key non-invasive tool for the detection of obstructive coronary artery disease (CAD) but has had limited investigation in the assessment of microvascular function. Recently, a new dual sequence automated in-line perfusion mapping has been developed allowing free breathing acquisition and pixel-wise quantification of myocardial blood flow (MBF) [10], overcoming previous limitations of semi-quantitative first-pass perfusion images with relative contrast uptake measures. This novel technique has demonstrated good performance in the detection of obstructive CAD as well as microvascular disease in patients with angina when validated with cardiac catheterization, as well as its ability to distinguish microvascular disease from multivessel coronary artery disease [11]. The aim of this study was to assess measures of CMD in obese patients with HFpEF using this novel fully quantitative myocardial perfusion mapping CMR technique and determine their associations with measures of diastolic dysfunction.

## Methods

The study was approved by the Institutional Review Board of Virginia Commonwealth University and all participants provided witnessed informed consent. Patients between the ages of 21 and 80 years with obesity (BMI > 30 kg/m^2^) and with stable symptoms of heart failure (NYHA class II-III), preserved left ventricular EF (> 50%) and without obstructive CAD were prospectively recruited at Virginia Commonwealth University Health System. Patients underwent transthoracic echocardiogram to confirm diagnosis of HFpEF defined as left ventricular EF > 50%, and evidence of diastolic dysfunction as evident by at least two of the following criteria defined by the American Society of Echocardiography guideline [12]: average E/e’ > 14, septal e’ velocity < 7 cm/s or lateral e’ velocity < 10 cm/s, tricuspid regurgitation velocity > 2.8 m/s, left atrial volume index > 34 ml/m^2^. We selected e’ as a measure of lusitropy and E/e’ as a surrogate for left ventricular filling pressures, as the preferred markers of diastolic dysfunction. All participants then underwent a pharmacologic stress perfusion CMR.

We excluded patients with obstructive CAD (by means of invasive or non-invasive coronary angiography and/or provocative test for myocardial ischemia), prior myocardial revascularization (coronary artery bypass surgery, percutaneous coronary intervention), prior myocardial infarction, severe valvular heart disease, contraindications to CMR, or estimated glomerular filtration rate < 30 ml/min/1.73 m^2^.

### Stress perfusion CMR image acquisition and analysis

All patients underwent stress perfusion CMR on a 1.5 Tesla system (Magnetom Aera, Siemens Healthcare, Erlangen, Germany). Scans were performed in accordance with local protocol, and patients were asked to refrain from caffeine for at least 12 h before the scan. Basal, mid-ventricular, and apical short-axis myocardial perfusion images were acquired both at rest and during hyperemia. Hyperemia was induced using adenosine infused via a peripheral cannula at a rate of 140 µg/kg/min for 3 min or a single bolus injection of regadenoson in one patient. Image acquisition was performed over 60 heartbeats with a bolus of 0.05 mmol/kg gadoterate meglumine (Dotarem, Guerbet SA, Paris, France) administered at 4 ml/s followed by a 20-ml saline flush during acquisition of the perfusion sequence.

We used a quantitative perfusion mapping sequence with an automated pixel-wise map generated in-line using Gadgetron reconstruction [10]. Quantitative perfusion imaging was performed with a dual sequence and single gadolinium (Gd) injection to estimate the arterial input function (AIF) and quantitative perfusion map as previously described [10]. Myocardial blood flow was estimated from the AIF and myocardial pixel time series [Gd] values. Average MBF (mL/min/g) was assessed per coronary artery territory according to the 17-segment model, excluding the apical segment. Global MBF was calculated by averaging MBF across the 3 slices. Myocardial ischemic burden was defined as percentage of myocardial segments with stress MBF less than 1.94 mL/min/g [11]. The 16-segments were further sub-divided transmurally to create subendocardial and subepicardial segments with corresponding MBF values and ratios to reflect transmural gradient. Global endocardial:epicardial (endo:epi) ratio was calculated by averaging the ratio across the 3 slices. Relative subendocardial hypoperfusion was defined as endo:epi perfusion ratio < 1 during hyperemia [13, 14]. Visual assessment for myocardial perfusion defects was performed by an independent expert operator blinded to the results of the quantitative perfusion maps.

Additional CMR imaging included a breath-held steady state free precession short-axis cine stack covering the LV and parametric mapping for extracellular volume (ECV) acquired and analyzed in accordance with guidelines [15, 16]. Briefly, endo- and epicardial cine contours were created using a semi-automated algorithm with manual adjustments in cvi^42^ software (Circle Cardiovascular Imaging Inc., v5.11.4, Calgary, Canada) for delineation of LV volumes and calculation of LV EF by Simpson’s rule [15]. Short-axis MOLLI T1 maps were acquired before and 15 min after contrast for ECV quantification in cvi^42^ software; endocardial and epicardial contours were manually applied with a 10% erosion offset to minimize partial volume effects from the blood pool and/or epicardial fat [16].

### Statistical analysis

Continuous variables were expressed as median (interquartile range) and categorial variable as number (percentage) for potential deviation from the Gaussian distribution. The non-parametric Spearman’s rank test was used for correlations between two variables. The McNemar Chi-Square test was used to compare discrete variables in paired samples. A p value less than 0.05 was considered significant. All the analyses were completed using SPSS, version 24.0 (SPSS; Chicago, IL).

## Results

We enrolled 21 patients between July 2020 to December 2020 and March 2021 to November 2021, with interruptions in recruitment related to COVID-19 pandemic. Two patients were excluded after CMR: one due to new diagnosis of hypertrophic cardiomyopathy that became evident on CMR, and one due to severe perfusion abnormality suggestive of epicardial CAD and later confirmed by cardiac catheterization. Thus, a total of 19 patients met the enrollment criteria and were included in the analysis. All 19 (100%) patients were female and 8 (42%) were self-referred Black or African American. Age was 64 years [56–72] and BMI was 34 kg/m^2^ [31–41]. Angina was present in 10 (53%) of the patients. Medications at the time of enrollment are summarized in Table 1. Majority of the patients (74%) were on statin therapy, 47% on beta-blockers and 47% on calcium channel blockers. Clinical characteristics of the patients are summarized in Table 1.

**Table 1:** Baseline characteristics of the population

	Population (n = 19)
**Demographics**	
Female (n, %)	19 (100%)
Age, years (median, IQR)	64 [56–72]
Black or Afro-American (n, %)	8 (42%)
Body mass index, kg/m^2^ (median, IQR)	34 [31–41]
**Past medical history**	
Tobacco	
Current use (n, %)	1 (5%)
History of use (n, %)	6 (32%)
Alcohol consumption (n, %)	4 (21%)
Hypertension (n, %)	18 (95%)
Diabetes mellitus (n, %)	9 (47%)
Hypercholesterolemia (n, %)	18 (95%)
Coronary artery disease (n, %)	0 (0%)
Atrial fibrillation (n,%)	0 (0%)
Chronic kidney disease stage III-V (n,%)	0 (0%)
COPD (n,%)	1 (5%)
PM/ICD (n,%)	0 (0%)
**Clinical presentation**	
NYHA class II-III (n, %)	19 (100%)
Angina (n,%)	10 (53%)
**Laboratory**	
hsCRP, mg/L (median, IQR)	4.22 [2.45–8.14]
NTproBNP, pg/mL (median, IQR)	55 [50–163]
IL-6, pg/mL (median, IQR)	2.9 [2.5–4.4]
**Vital signs**	
Systolic blood pressure, mmHg (median, IQR)	134 [118–148]
Diastolic blood pressure, mmHg *(*median, IQR)	65 [56–76]
Heart rate, beats/min (median, IQR)	70 [64–78]
**Echocardiogram – Diastolic function**	
E/A ratio (median, IQR)	0.88 [0.70–0.90]
Septal e’, cm/s (median, IQR)	6 [5-7]
Lateral e’, cm/s (median, IQR)	7 [6-8]
E/e’ ratio (median, IQR)	10.9 [9.9–12.8]
Left atrial volume index, ml/m^2^ (median, IQR)	35 [30–38]
**Medications at enrollment**	
ACEi/ARBs	9 (47%)
Beta-Blockers	9 (47%)
Aspirin	8 (42%)
Statin	14 (74%)
Calcium channel blockers	9 (47%)
SGLT-2i	8 (42%)
Loop diuretics	7 (47%)
MRA	6 (32%)

Abbreviations: ACEi (angiotensin converting enzyme inhibitor), ARB (angiotensin II receptor blocker), COPD (chronic obstructive pulmonary disease), hsCRP (high sensitivity c-reactive protein), ICD (implantable cardiac defibrilator), IL-6 (interleukin 6), MRA (mineralcorticoid receptor antagonist), NT-pro BNP (N-terminal pro B-type natriuretic peptide), NYHA (New York Heart Association), PM (pacemaker), SGLT-2i (sodium glucose cotransporter-2 inhibitor)

CMR characteristics of the patients are summarized in Table 2. Left ventricle EF was 71% [66-73.5%]. All subjects had an abnormal subendocardial perfusion pattern with global stress endo:epi ratio of < 1.0 (0.87 [0.81–0.90]). Global stress MBF was 2.43 ml/min/g [2.16–2.78] and regional stress MBF was 2.58 ml/min/g [2.23–2.84] in the left anterior descending coronary artery territory, 2.15 ml/min/g [1.94–2.52] in the right coronary artery territory and 2.33 ml/min/g [2.07–2.61] in the circumflex coronary artery territory. Global MPR was 2.34 [2.07–2.88]. There was no significant correlation between global stress MBF and global extracellular volume (R = 0.114, p = 0.663).

**Table 2:** Cardiac MRI parameters

	**Population (n = 19)**
Left ventricle ejection fraction, % (median, IQR)	71 [66-73.5]
Indexed LVEDV, mL/m^2^ (median, IQR)	69.8 [61.9–74.6]
Indexed LVESV, mL/m^2^ (median, IQR)	20 [16.5–23.3]
Indexed LV mass, g/m^2^ (median, IQR)	52.2 [48.8–58.5]
Indexed cardiac output, L/min/m^2^ (median, IQR)	3.4 [2.9–3.6]
Extracellular volume, % (median, IQR)	29.9 [27.7–33.4]
Stress global MBF, mL/min/g (median, IQR)	2.43 [2.16–2.78]
Global MPR	2.34 [2.07–2.88]
Stress global Endo:Epi ratio	0.87 [0.81–0.90]
Total ischemic burden, % (median, IQR)	6% [0-34.4]

Abbreviations: Endo (sub-endocardial), Epi (sub-epicardial), LVEDV (left ventricular end diastolic volume), LVESV (left ventricular end systolic volume, MBF (myocardial blood flow), MPR (myocardial perfusion reserve)

**Fig. 1:**
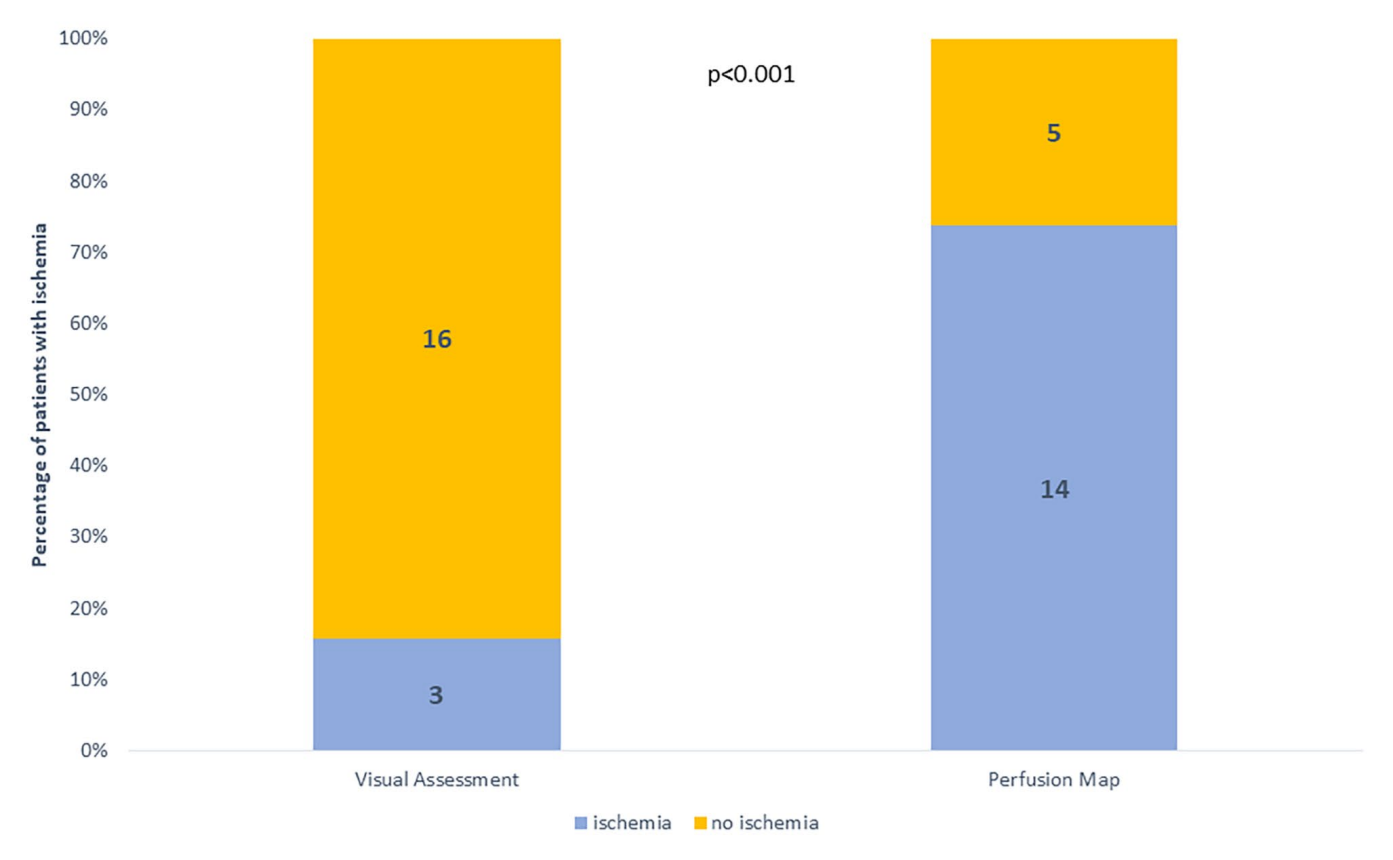
Use of quantitative perfusion map results in detection of more patients with myocardial hypoperfusion

By quantitative perfusion analysis, segmental myocardial hypoperfusion was detected in 14 (74%) of the patients with a median ischemic burden of 6% [0-34.4]. In contrast, abnormal perfusion by visual assessment was detected in only 3 (16%) of the patients (p < 0.001 comparing visual assessment to quantitative assessment) (Fig. 1). We found a statistically significant correlation between global stress MBF and total IB (R=-0.866, p = < 0.001) as well as the endo:epi ratio with IB (R=-0.510, p = 0.026)

**Fig. 2:**
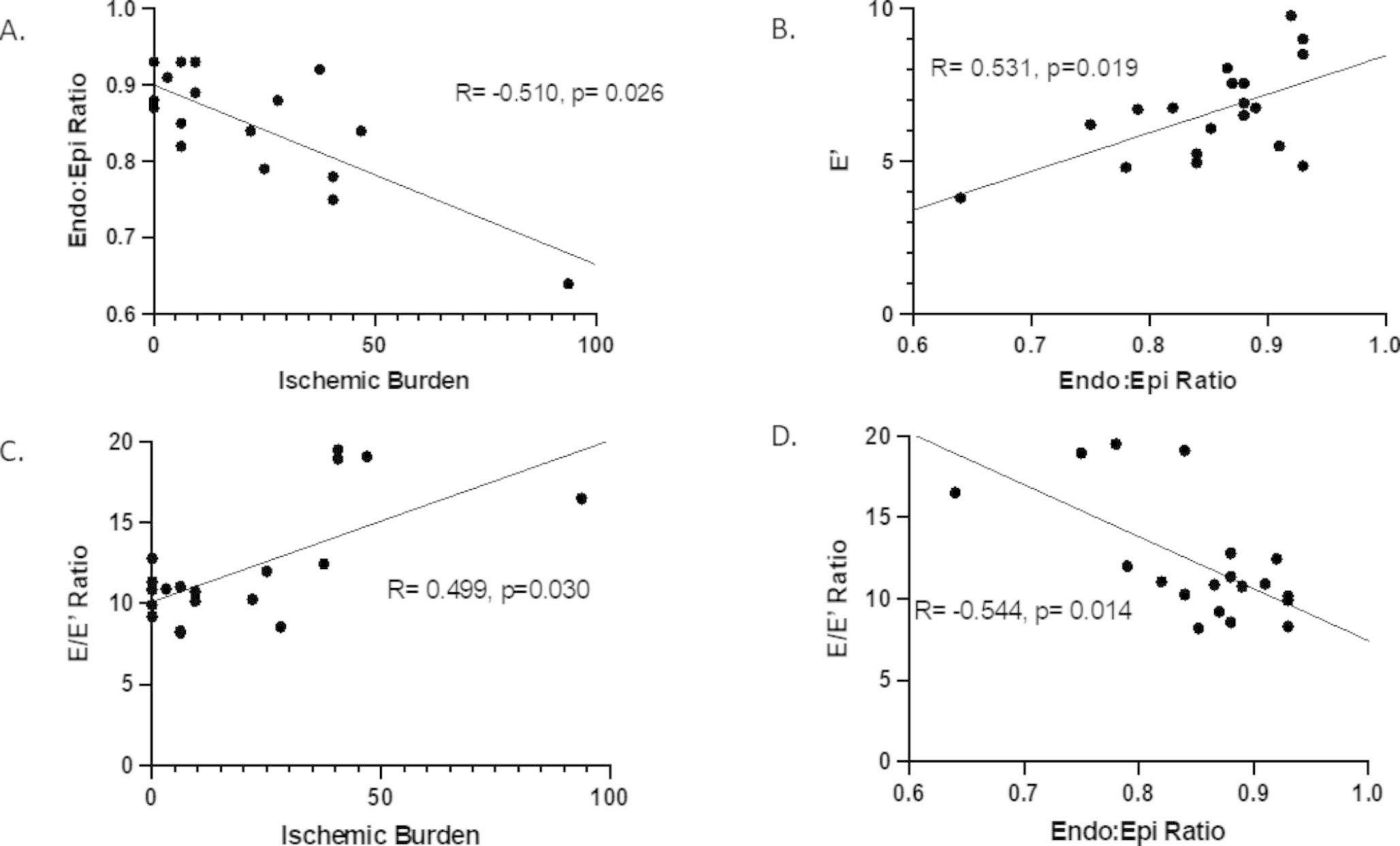
Measures of perfusion correlate with diastolic dysfunction. Lower subendocardial perfusion correlates with higher total ischemic burden (A). The subendocardial hypoperfusion correlates with E’ velocity (B) a measure of lusitropy. Both the abnormal subendocardial perfusion ratio and the total ischemic burden correlate with E/E’ (C,D) a measure of left ventricular filling pressure

**Fig. 3:**
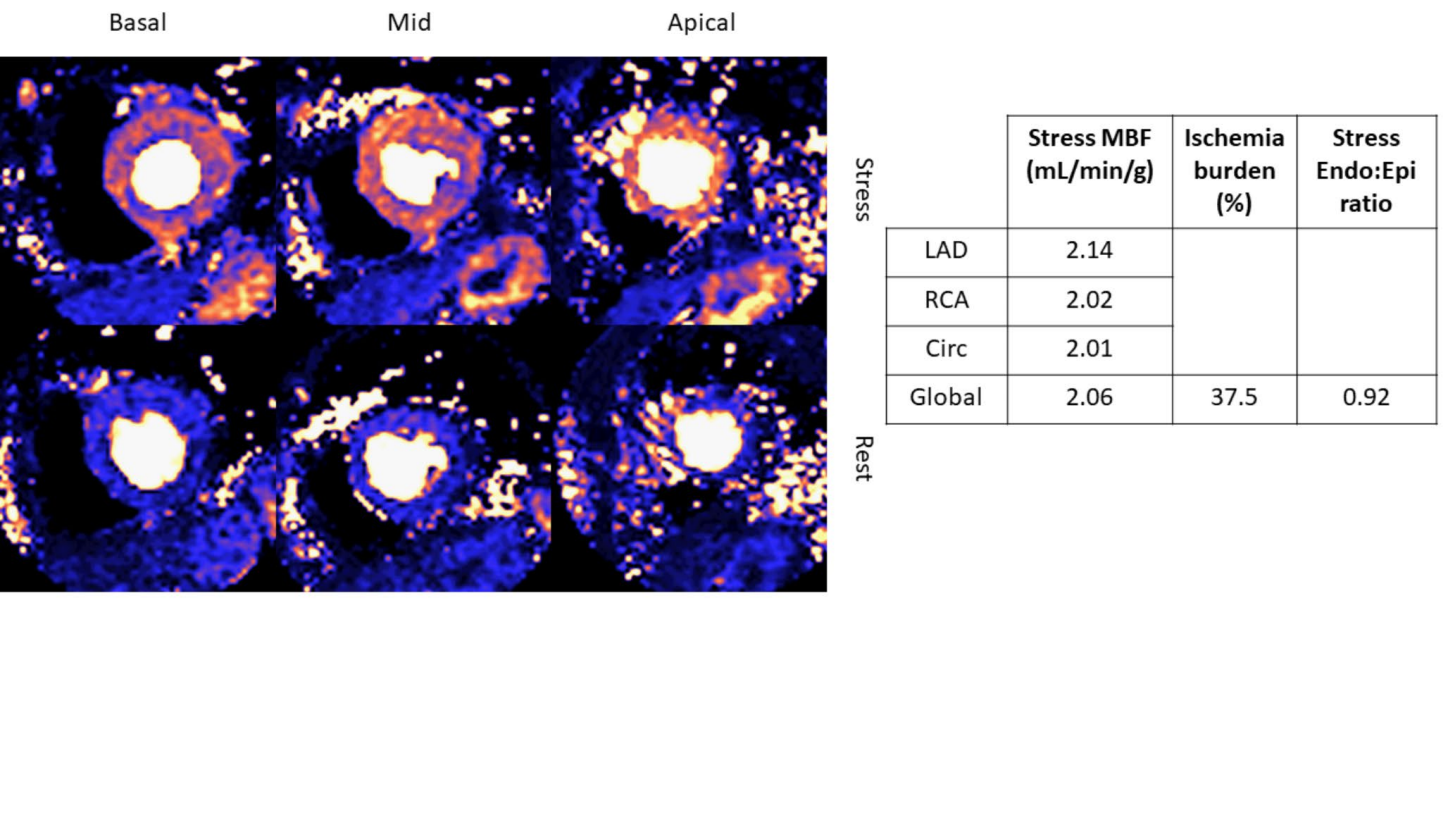
An example of a stress-perfusion CMR in a patient with HFpEF and angina in which a previously invasive coronary angiography documented angiographically normal coronary arteries. Basal, mid-ventricular, and apical short-axis perfusion images were acquired both at rest and during hyperemia to obtain a perfusion map using an automated quantitative myocardial perfusion map. No visual inducible defect was noted. Global stress MBF was reduced at 2.06 mL/min/g. There was abnormal subendocardial perfusion with subendocardial-to-subepicardial ratio of 0.92. The total myocardial ischemic burden was 37.5%

Doppler echocardiography demonstrated septal e’ 6 [5-7] cm/s, lateral e’ 7 [6-8] cm/s, average e’ 6.7 [5.25–7.55] cm/s, E/e’ ratio average 10.9 [9.9–12.8]. There was a significant correlation between global stress endo:epi perfusion ratio on CMR with diastolic dysfunction as measured by average e’ (R = 0.531, p = 0.019) and E/e’ (R=-0.544, p = 0.014) (Fig. 2). Total IB also correlated with E/e’ (R = 0.499, p = 0.030) a surrogate marker for LV filling pressure. An example of a stress myocardial perfusion map of a patient with HFpEF is represented in Fig. 3.

## Discussion

Using a fully quantitative non-invasive CMR assessment of microvascular disease, in a cohort of women with obesity and HFpEF without obstructive CAD, the current study shows that: (1) all patients had an impaired stress endo:epi perfusion ratio (< 1.0); (2) the endo:epi ratio correlated with echocardiographic measures of diastolic dysfunction (E/e’ ratio and e’ velocity); and (3) the great majority of patients (74%) had evidence of regional hypoperfusion with median ischemic burden of 6%, also correlating with higher E/e’ ratio, a surrogate for elevated left ventricular filling pressures.

CMD is common in patients with HFpEF, and a decrease in myocardial blood flow has been shown to be associated with higher incidence of mortality and adverse cardiovascular outcomes independent of traditional risk factors [17, 18]. It has been proposed that in patients with CMD the pre-arteriolar constriction in the sub-epicardium causes a pressure drop which reduces blood flow in the subendocardial layers and creates transmural blood flow steal in response to epicardial arteriolar dilation [19]. Patients with CMD have lower stress endo:epi ratio and higher incidence of ischemia than those with normal coronary flow reserve [14]. Our study demonstrates that CMD is common in women with obesity and HFpEF as demonstrated by reduced endo:epi perfusion ratio. In this cohort all subjects had evidence of absolute reduction in subendocardial perfusion with stress which was one standard deviation below the reported mean of 1.05 in a normal cohort [14]. This abnormal subendocardial perfusion also correlated with total ischemic burden which is a measure of total number of myocardial segments with reduced blood flow.

Ischemia and myocardial injury have been thought to be the underpinning of hemodynamic derangement in patients with HFpEF, and CMD may promote myocyte injury, myocardial fibrosis, and stiffening in pathogenesis of HFpEF [4]. Myocardial injury as evident by elevated troponin levels with increase in oxygen demand has been associated with higher filling pressures and decreased cardiac reserve [20]. Additionally, a reduction in coronary perfusion during vasodilator hyperemia in CMD has been shown to disproportionately affect the subendocardial blood flow [14]. In our cohort the reduced subendocardial perfusion also correlated with lower e’ velocity, a measure of myocardial lusitropy, and with higher E/e’ ratio, a surrogate marker of LV filling pressure. This finding suggests that relative subendocardial hypoperfusion may play an important role in development of diastolic dysfunction and the clinical syndrome of HFpEF.

Ischemia caused by CMD may have a patchy distribution and may not be confined to a singular vascular bed [19]. The unevenly distributed ischemia may be compensated for by increased flow of the interposed unaffected regions. Multiple different noninvasive modalities can be used to assess myocardial ischemia; however cardiovascular imaging tests designed to detect later stages of ischemia cascade such as abnormal wall motion or reduced epicardial coronary perfusion are not adequately sensitive to detect this patchy distribution of myocardial ischemia from coronary microvascular abnormality in the earlier stages of ischemia [14, 21]. In our cohort, not only did all subjects had evidence of global subendocardial hypoperfusion, two thirds were also found to have segmental myocardial hypoperfusion using the quantitative assessment. Fully quantitative myocardial perfusion assessment using CMR allows for detection of microvascular dysfunction with high specificity (70–90%) and it has been shown to be superior to visual assessment [11, 21]. In our study we also demonstrated that quantitative assessment identified more patients with hypoperfusion compared to visual assessment as 74% of the patients were found to have some degree of ischemic burden as detected by quantitative assessment, while visual assessment failed to identify any perfusion abnormality in 84% of the cases. This highlights the importance of a fully quantitative non-invasive technique in assessment of CMD as the disease process involves the microvasculature which is beyond the visual detection. Total ischemic burden also correlated with E/e’ where patient with higher ischemic burden also had higher filling pressures.

HFpEF is a heterogenous clinical syndrome in which cardiovascular risk factors culminate in heart failure symptoms. In our study we have demonstrated that CMD is present in obese women with HFpEF and can be measured non-invasively using a novel CMR perfusion technique. It is difficult to determine to what extent the perfusion abnormalities are related to the underlying comorbidities; nonetheless, reduced myocardial perfusion is a marker of higher cardiovascular risk and associated with death and heart failure hospitalization [22]. The relationship between diminished coronary reserve and impaired diastolic function may underlie the poorer prognosis and CMD may precede clinical heart failure symptoms. There are currently no disease modifying therapies targeting microvascular perfusion in patients with HFpEF. Whether improving the microvascular blood flow can improve diastolic function remains to be explored in future studies.

We do recognize that there are limitations to our study. First, we recognize that the sample size is small and all patients were female, although not by design; while a female predominance in CMD and HFpEF is expected this is a potential limitation of this study. Nevertheless, the results of this pilot study may help develop future larger trials in the pathophysiology of heart disease in women, a condition that is too often neglected, and in a field of medicine where underrepresentation of women is often the prevailing problem. We also recognize that there was no invasive coronary assessment for CMD to validate our measures of MBF. Our study is, however, consistent with findings of others using quantitative non-invasive assessment of MBF. Additionally, adenosine is shown to detect non-endothelial-dependent coronary flow changes, and therefore we cannot completely rule out endothelial-dependent mechanism of abnormal coronary flow [23]. Finally, due to novelty of this technique, there is limited external validity of this technique to define differences of MBF due to age, sex or comorbidities making it difficult to define generalizable cut off values for CMD.

In conclusion, we have demonstrated that coronary microvascular dysfunction is common in women with obesity and HFpEF as demonstrated by abnormal stress endocardial-to-epicardial perfusion ratio using a novel fully automated quantitative perfusion mapping with cardiovascular magnetic resonance. The great majority of the patients also had evidence of regional hypoperfusion which correlated with abnormal transmural perfusion gradient. Abnormal sub-endocardial perfusion and regional hypoperfusion also correlated with measures of diastolic dysfunction.
